# Dissociating voluntary mental imagery and mental simulation: Evidence from aphantasia

**DOI:** 10.3758/s13421-025-01731-y

**Published:** 2025-06-10

**Authors:** Laura J. Speed, Emma M. E. Geraerds, Ken McRae

**Affiliations:** 1 Centre for Language Studies, Radboud University, Nijmegen, Netherlands; 2 Department of Psychology, University of Western Ontario, London, Canada; 3 Donders Institute for Brain, Cognition, and Behaviour, Radboud University, Nijmegen, Netherlands

**Keywords:** Aphantasia, Mental simulation, Mental imagery, Embodiment

## Abstract

Intentional visual imagery is a component of numerous aspects of cognition. Related to visual imagery, mental simulation plays a role in language comprehension: modality-specific regions of the brain are activated as an implicit part of people understanding language. The degree of overlap between the processes underlying conscious, voluntary visual imagery versus less conscious, more automatic mental simulation is unclear. We investigated this issue by having aphantasics (people who are unable to experience conscious voluntary visual imagery) and control participants perform a property verification task in which they were asked whether a property is a physical part of an object (e.g., is *mane* a physical part of a *lion*?). We manipulated the false trials so that the two words either were associated (semantically related) but did not form an object–part combination (*monkey*–*banana*), or were not associated (*apple*–*cloud*). Solomon and Barsalou (*Memory & Cognition, 32*, 244–259, 2004) demonstrated that word association influenced responses when the words in the false trials were not associated, whereas when they were associated, perceptual measures most strongly influenced the results, indicating mental simulation. In the present study, control participants and aphantasics demonstrated similar evidence of the use of both mental simulation and word association when verifying whether the words formed an object–part combination. These results suggest that visual imagery and mental simulation are at least somewhat separable cognitive processes.

**Supplementary Information:**

The online version contains supplementary material available at 10.3758/s13421-025-01731-y.

Humans can voluntarily and purposefully create internal visual images in their mind, known as visual imagery. Visual imagery is critically involved in many cognitive tasks. For example, it has a role in working memory (Keogh & Pearson, 2011), autobiographical memory (Anderson et al., 2017; Greenberg & Knowlton, 2014; Sheldon, 2022), and event future thinking (D’Argembeau & Van Der Linden, 2004, 2012). Visual imagery is also thought to be recruited when comprehending language. However, there is an ongoing debate as to whether language comprehension uses the same voluntary, conscious imagery processes as those used when one is explicitly asked to “Imagine a . . . ,” or whether a separate, less conscious, more automatic process, known as mental simulation, is used (Mak & Faber, 2024; Muraki et al., 2023b).

Evidence from neuroimaging shows that voluntary visual imagery recruits regions of the brain involved in veridical visual perception, including early visual areas (Albers, et al., 2013; Slotnick et al., 2005; Stokes et al., 2009). For example, retinotopic maps constructed via fMRI were similar when participants viewed a flickering checkerboard and when they imagined one (Slotnick et al., 2005). Mental images of works of art can even be decoded from low-level visual activation in the brain (Naselaris et al., 2015). Visual brain regions are also activated when comprehending language (Mak et al., 2023; Pulvermüller & Hauk, 2006; Saygin et al., 2010; Simmons et al., 2007; van Dam et al., 2012). For example, performing property judgments about color on word pairs (e.g., *TAXI* > *yellow*) activates brain regions recruited during color perception (Simmons et al., 2007). In addition, reading sentences about motion (e.g., *The wild horse crossed the barren field*) activates motion-sensitive visual areas of the brain (Saygin et al., 2010). This evidence is consistent with embodied theories of language comprehension that propose that activation of modality-specific regions of the brain takes place during language comprehension (Barsalou, 2008; Meteyard et al., 2012). This perceptual activation is known as mental simulation. It is important to note that there exist inconsistencies and overlap regarding terminology within and across fields. Researchers such as Schacter and Addis (2007) use the term “simulation” when describing how people voluntarily and explicitly generate imagery in autobiographical memory and event future thinking tasks. In this article, to remain consistent with the language comprehension literature, we use “mental simulation” to denote the more implicit automatic process that is part of understanding language.

Although there are similarities between visual imagery and the visual simulation that occurs during language comprehension, it remains unclear to what extent the two processes overlap (Mak & Faber, 2024; Muraki et al., 2023b). Mental simulation has been described as less conscious (Connell & Lynott, 2016; Muraki et al., 2023b; Zwaan & Pecher, 2012) and more schematic than visual imagery (Barsalou, 1999). While Barsalou (1999) acknowledges that mental simulation can sometimes produce “conscious counterparts” (p. 583), the basic definition is that unconscious mental images underlie mental simulation. Supporting this proposal, Connell and Lynott (2016) showed that information is lost when the contents of mental simulations are transferred to conscious imagery. Mental simulation is also considered to be automatic and involuntary. For example, activation of visual representations during language comprehension has been found to occur as early as 200 ms after word onset (Ostarek & Huettig, 2017). Although it has been proposed that mental simulation uses a subset of the mechanisms involved in mental imagery (Schendan & Ganis, 2012), the evidence concerning the degree to which mental imagery and mental simulation are related is mixed. On the one hand, individual differences in specific forms of motor imagery correlate with effects of sensorimotor simulation in some language tasks (Cayol et al., 2020; Muraki et al., 2023a; Muraki & Pexman, 2021). Furthermore, strong visual imagers have been found to have better long-term memory for words with more sensorimotor associations (McKelvie & Demers, 1979).

On the other hand, there is evidence to suggest that mental simulation and mental imagery are distinct processes. Willems et al. (2010) found that areas of the brain activated during a mental simulation task were distinct from regions activated by an explicit mental imagery task. However, Willems et al. (2010) specifically addressed motor imagery, which may differ from sensory imagery. In other studies, individual scores on visual and auditory imagery questionnaires do not predict performance in mental simulation tasks (Pecher et al., 2009; Speed & Majid, 2018; Zwaan & Pecher, 2012). It is possible, however, that the range of imagery abilities in these samples was not sufficiently large to detect effects of individual differences in imagery on mental simulation processes. In addition, it is likely that most participants were able to visualize to some extent.

## Aphantasia

A more direct way to establish the extent to which mental simulation and conscious, voluntary visual imagery are related is to investigate individuals with no conscious experience of visual imagery—that is, with aphantasia (Muraki et al., 2023a, b). Aphantasia is a condition in which people are unable to experience conscious voluntary visual imagery (Zeman, 2024; Zeman et al., 2015), and often imagery in other sensory modalities as well (Dawes et al., 2024). Aphantasia occurs in approximately 4% of the population (Dance et al., 2022), and has been shown to affect other cognitive processes, such as autobiographical memory and event future thinking (Dawes et al., 2020, 2022; Milton et al., 2021). If visual imagery underlies language comprehension, it is puzzling why people with aphantasia do not report difficulties with understanding spoken and written language.

Initial evidence suggests that aphantasics may have reduced mental simulation during reading compared with controls. Aphantasics have a reduced emotional response to frightening stories, but not frightening images (Monzel et al., 2023; Wicken et al., 2021), which could be due to a lack of mental simulation. Aphantasics also failed to show motor simulation, measured with motor-evoked potentials (MEPs) when reading manual action sentences (e.g., “I have a hair on my arm, I pull it out”; original stimuli presented in French: “J’ai un cheveu sur mon bras, je le retire” ).With longer texts, aphantasics appear to be impaired in deep reading—operationalized as the ability to select the best-fitting words for a sentence context (Dupont et al., 2023). Aphantasics also report being less absorbed in a short story and less transported to the story world compared with controls (Speed et al., 2024). However, this type of becoming immersed in a story may involve conscious visual imagery rather than mental simulation. It therefore remains unclear whether aphantasics mentally simulate during language comprehension.

The current evidence for whether people with aphantasia can engage in involuntary unconscious imagery, which may be more related to mental simulation, is mixed. Evidence from the binocular rivalry paradigm suggests that people with aphantasia are impaired in both voluntary, conscious, and involuntary, unconscious imagery (Keogh & Pearson, 2021; Purkart et al., 2025), and it has been argued that aphantasia should not be characterized as solely a specific deficit in voluntary imagery (Krempel & Monzel, 2024). Keogh and Pearson (2021) found that aphantasic participants’ attention to visual features (green vertical or red horizontal lines of a plaid stimulus) was affected by an attentional cue (being cued to attend to green or red lines of a prime image), but the same attentional cue did not activate visual representations in the form of attentional templates—thought to unconsciously activate visual representations—like it did in control participants. Similarly, Purkart et al. (2025) found no evidence of binocular rivalry when participants were implicitly primed to imagine a Gabor patch via associations between colors and Gabors learned in an association phase. There is also evidence that aphantasics do not activate motor simulation, measured via MEPs, during implicit action observation, in addition to explicit motor imagery (Dupont et al., 2024).

On the other hand, some evidence suggests that involuntary, unconscious imagery is possible in aphantasia. In a case study conducted by Duan et al. (2024), an aphantasic participant did not differ from controls on a differential threshold task involving involuntary imagery. Participants viewed pairs of visual gratings consecutively and had to judge whether the second grating had rotated clockwise or counterclockwise to the first grating. Before presentation of the first grating, a high- or low-pitch tone cued the orientation of the second grating. In the involuntary imagery condition, an association between the tones and the angle of the second grating had been learned implicitly. Interestingly, the aphantasic also did not differ from controls when explicitly asked to imagine the orientation of the second grating when the interval between the instruction to imagine and presentation of the grating was very short (500 ms), but they performed worse than controls with a longer interval (6,000 ms). Since it has been suggested that voluntary imagery can only affect perception with longer durations of imagery (Pearson et al., 2008), these results can be interpreted as showing an impairment in voluntary but not involuntary imagery.

Neuroimaging methods have also been used to reveal the potential for involuntary imagery in aphantasia. Using fMRI and multi-voxel pattern analyses (MVPA), Cabbai et al. (2024) found that voluntary, conscious visual imagery elicited via evocative sounds (e.g., the sound of a crowd of seagulls) could be decoded from representations in the primary visual cortex (V1) for control participants, but not for aphantasics. Interestingly, when aphantasics passively listened to the same sounds, decoding accuracy in V1 was greater than chance, even though participants did not report any conscious imagery. Whilst it is unclear from this finding if unconscious imagery in aphantasia could play a role in cognition, it at least highlights the potential role it has.

Another possibility is that aphantasics are capable of both voluntary and involuntary imagery, but have no conscious access to it. Liu and Bartolomeo (2023) asked aphantasics, typical imagers and vivid imagers to imagine pairs of objects, faces, and spatial layouts and make judgments about them (e.g., “beaver,” “fox,” “which is longer?”). Surprisingly, aphantasics showed no impairment in their accuracy performing the task, but their response time was slower for all domains except space. The authors suggest that the aphantasics were able to generate mental imagery sufficient to perform the task, but may have a deficit in phenomenal consciousness, which affects speed of information processing. Similarly, Jacobs et al. (2018) found that an aphantasic individual did not differ from controls on a working memory task putatively requiring visual imagery, but they did lack insight into their performance, in line with a deficit in phenomenal consciousness. On the other hand, their performance did decline when the task required a high level of precision, which could mean that the easier levels of the task were completed by nonvisual compensatory strategies.

While the existing evidence for the presence of involuntary imagery in aphantasia is mixed, Krempel and Monzel (2024) argue that involuntary imagery should not be considered a unified kind and may instead come in multiple forms. From this perspective, involuntary imagery elicited while comprehending language (i.e., mental simulation) may be a different kind relative to the involuntary imagery elicited in the tasks outlined above.

## The present study

Our aim is to test the extent to which individuals with aphantasia engage in mental simulation as part of language comprehension. We investigated the presence of mental simulation in aphantasia using an established property verification task previously used to demonstrate mental simulation. Solomon and Barsalou (2004) conducted an experiment to test the language and situated simulation (LASS) theory (Barsalou et al., 2008). According to LASS, linguistic associations between words are sufficient to perform shallow semantic tasks, and are activated early in the time course of language processing (see also Connell, 2019; Louwerse, 2011). In contrast, mental simulation is more useful during deep semantic tasks, and is delayed relative to the influence of linguistic associations.

In Solomon and Barsalou’s (2004) property verification task, participants decided whether a presented property was a physical part of an object (e.g., is *nose* a part of *face*?). On false trials (when the property was not a physical part), property-concept pairs were manipulated to have either strong word association (associated condition e.g., *apple*–*pear*) or no word association (unassociated condition e.g., *apple*–*cloud*). False-trial word association was manipulated between participants. Property verification times to true trials were faster when the false trials were unassociated compared with when they were associated. This suggests that participants used a rapidly unfolding word association strategy to distinguish between associated true concept–property pairs and completely unassociated word pairs. In contrast, when false trials were associated, word association was no longer helpful and participants had to use mental simulation, a slower process, to decide whether the two words formed a concept–property pair. Perceptual and linguistic ratings of the concept–property pairs also predicted responses. When false trials were associated, perceptual ratings predicted performance (response time and accuracy), but when false trials were unassociated, linguistic ratings did. Follow-up work with fMRI found that visual association cortex was activated when false trials were associated but not when they were unassociated, further supporting the initial conclusion that mental simulation occurred (Kan et al., 2003). Overall, their results suggest that the influences of linguistic association and mental simulation vary depending on task context.

In the present study, we used Solomon and Barsalou’s (2004) design to compare performance on the property verification task between aphantasics and controls. If mental simulation and mental imagery are related, and therefore aphantasics do not engage in mental simulation, we expect aphantasics to be slower than controls in the associated condition in which performing the task via linguistic associations is unhelpful and mental simulation must be used. Furthermore, we expect perceptual ratings to predict performance in the associated condition for the controls but not for the aphantasics. We do not expect the groups to differ in the unassociated condition in which the task can be performed by linguistic association alone. If instead mental simulation and mental imagery are distinct, then aphantasics and controls should not differ. Both groups should have faster response times for true trials in the unassociated compared with the associated condition (because judgments via linguistic associations are fast). Furthermore, linguistic associations should predict performance in the unassociated condition in both groups, and perceptual ratings should predict performance in the associated condition in both groups.

## Method

The study was ethically approved by the Ethics Assessment Committee of the authors’ university and preregistered on the Open Science Framework: https://osf.io/9ntdy

### Participants

**Fig. 1:**
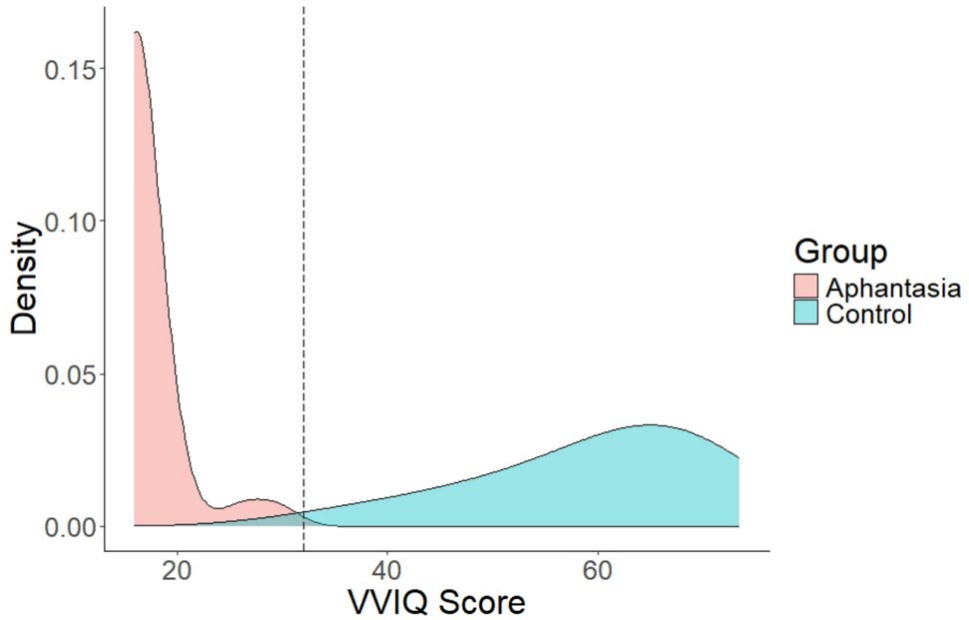
Distribution of Vividness of Visual Imagery Questionnaire (VVIQ; Marks, 1973) scores across final set of aphantasics and controls. A score of less than 32 indicates aphantasia, as indicated by the dashed line

All participants were native speakers of Dutch. Aphantasic participants were recruited via advertisements on social media groups dedicated to aphantasia (e.g., on Facebook and Reddit) and contact with researchers who were also conducting research with aphantasic participants. Control participants were recruited on Prolific. Participants were paid 5 euros for their participation either via Prolific or as an online shopping voucher. Several participants were removed from the dataset according to our preregistered data exclusion criteria. The Vividness of Visual Imagery Questionnaire (VVIQ; Marks, 1973) was used to determine whether or not participants were aphantasic. Three aphantasic participants were removed for having a score greater than 32, indicating intact visual imagery. Two control participants were removed for having a VVIQ score less than 32, indicating reduced vividness of visual imagery. Five participants were removed from all analyses for having an overall accuracy score of less than 80% correct (three aphantasics and two controls). The final sample consisted of 30 participants with aphantasia (*M*_age_ = 39.3 years, *SD* = 13.6) and 29 control participants (*M*_age_ = 36.3 years, *SD* = 13.2). The two groups did not differ in age, *t*(57) = 0.86, *p* = .4. Figure 1 displays the distribution of mean VVIQ scores across aphantasics and controls.

### Design

The study is a 2 × 2 between participants design with the factors group (aphantasic vs. control) and relatedness of the false trials (associated vs. unassociated).

### Stimuli

Concept–property pairs were selected based on Solomon and Barsalou’s (2004) stimuli and translated to Dutch. Some items needed to be changed because their Dutch translation did not work or because they were outdated (e.g., a *receiver* for a *phone*). There were 100 true pairs in which properties were physical parts (e.g., *bull*–*horns*). There were 100 false pairs in which the property was associated with the concept in one of three ways (associated condition): thematically related (e.g., *banana*–*chimpanzee*), related via taxonomic category (e.g., *donkey*–*mule*), or taxonomically related parts (e.g., *guitar*–*keyboard*). There were 100 false pairs in which the property and the concept were unrelated (unassociated condition). The unassociated pairs contained the same concepts and properties as the associated pairs but were re-paired ensuring that the concept and property were unrelated. There were also 72 practice pairs, with an equal number of true, false associated and false unassociated pairs.

### Predictors

All true pairs were rated by a separate set of participants on seven factors that were significant predictors of responses in Solomon and Barsalou (2004): L_conc_prop, P_area, P_left, P_initial, P_find, P_handle, and E_num_obj.^1^ The false pairs were also rated on L_conc_prop. L_conc_prop is a linguistic predictor that indicates the associative strength between the concept and property (ICC = .94). Participants were asked to rate how quickly they thought of the property word when they read the concept word on a scale of 1 (*didn’t think of it at all*) to 9 (*very fast*). The five factors starting with a P are perceptual predictors. For P_area (ICC = .93), participants indicated the area of the property as a percentage of the entire object (e.g., *what percentage of a bull is the horns?*, scale: 0 –100%). For P_left (ICC = .66), participants were asked to image a box around the internal visual image of the concept and indicate what percentage of the width of the box is the distance from the left edge of the box to the property as a percentage of the entire concept length (scale: 0–100%). For P_initial (ICC = .85), participants indicated whether or not the property was in their initial visual image of the concept (1 = yes, 0 = no). For P_find (ICC = .82), participants were asked how easily they could find the property in the visual image of the concept on a scale of 1 (*very difficult*) to 9 (*very easy*). For P_handle (ICC = .95), participants were asked to imagine the concept and rate the likelihood of touching the property on a scale of 1 (*not likely*) to 9 (*very likely*). The last factor, E_num_obj (ICC = .77), is an expectancy predictor of the amount of different objects that have the property. Participants were asked for each concept–property pair how many different sorts of objects have that property (1 = 1 object, 2 = 2–3 objects, 3 = 4–10 objects, 4 = 11–30 objects, 5 = 31–100 objects). As in the original study, this was used to control for the polysemy of the property words. For example, the property *nose* takes different forms for humans, animals, and planes. The reliability scores for each predictor were calculated using intraclass correlation in R (R Core Team, 2020).

### Procedure

The experiment was built with PsychoPy (Peirce et al., 2019) and hosted online with Pavlovia (pavlovia.org). The experimental procedure followed that of Solomon and Barsalou (2004). On each trial participants were shown the name of a concept for 500 ms, followed by a 1,200 ms interstimulus interval, and then the name of a property. The property remained on the screen until participants responded. Verification times were measured starting at the onset of the presentation of the property name and ending at the response. Participants were instructed to respond using the right arrow button for a “yes” if the property was a physical part of the concept, or the left arrow for “no” if it was not. They were instructed to use their dominant hand to respond and to answer as quickly as possible while keeping the number of errors as low as possible. After each answer, participants received feedback on whether or not their answer was correct (“correct” or “incorrect” appeared on the screen). Participants first received practice trials to become accustomed to the procedure. They were told there were 16 practice items. Note that 32 additional practice items were presented at the beginning of the block of experimental trials. Participants were given the opportunity to take a short break every 31 trials. After the experiment, participants were directed to a survey in Qualtrics (https://www.qualtrics.com) that included demographic questions and the VVIQ (Marks, 1973). The VVIQ scores were used to check participants’ visual imagery abilities and distinguish between the aphantasics and controls. The aphantasics were also asked additional questions about their experience with aphantasia, following Zeman et al. (2020).

## Results

Data and analysis scripts are available online (https://osf.io/j9znu/). Seventeen items were removed from analyses for having a mean accuracy of less than 70% (six true trials, 11 false associated). As preregistered, individual trials were removed if they fell outside of two standard deviations of a participants’ mean response time for correct true responses or correct false responses (6% of trials). In addition, response latencies shorter than 300 ms or longer than 3,000 ms were removed (a further 1%).

The data were analyzed using linear mixed effect models conducted in R (R Core Team, 2020) using the *lme4* package (Bates et al., 2015) with participants and items modelled as random intercepts. Separate models were conducted for true and false trials, for response time and accuracy. We tested the effect of group (aphantasic vs. control), relatedness (associated vs. unassociated), and the interaction between group and relatedness. Property word length and frequency (Zip frequency) were added to the models as control variables. We compared models with and without the factors of interest using likelihood ratio tests with chi-square.

### Response time

**Fig. 2:**
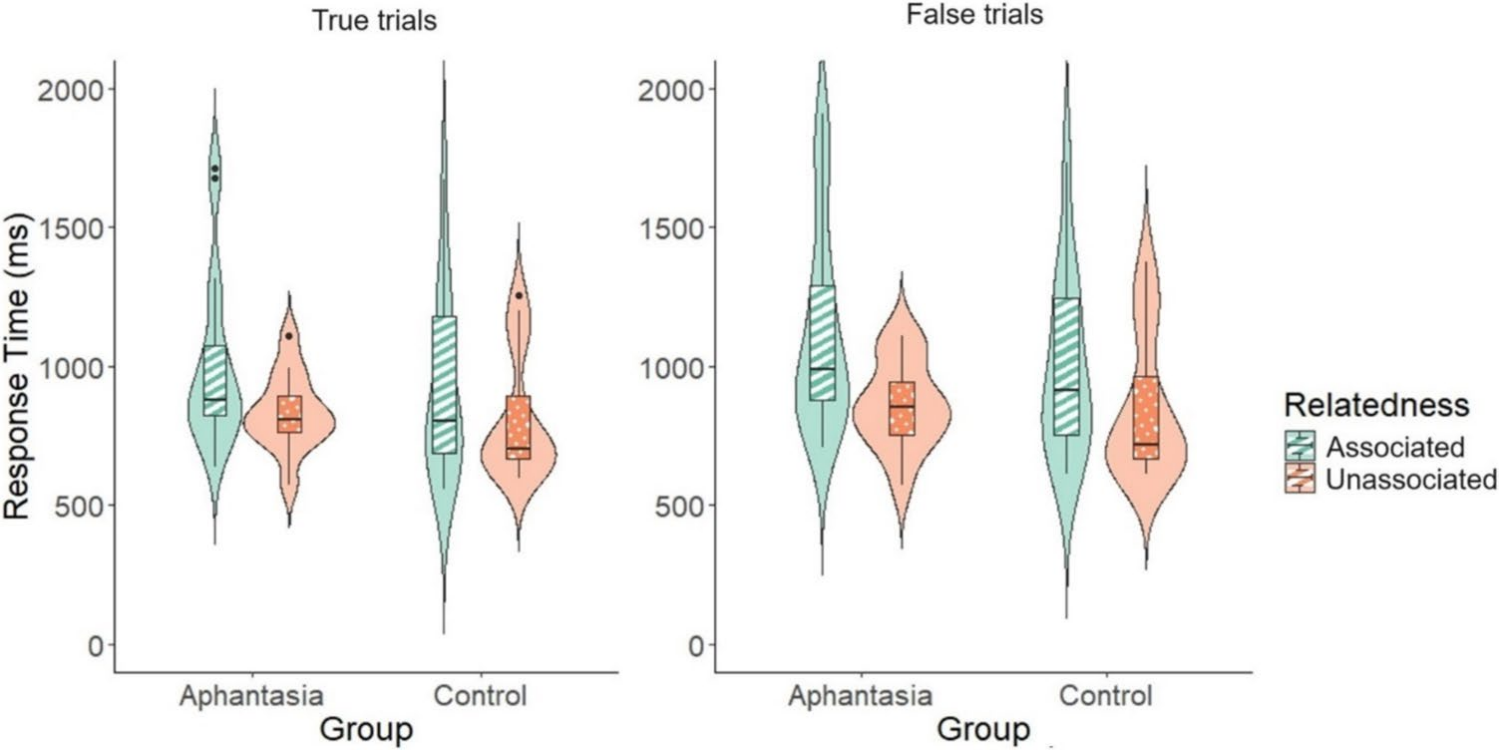
Response time (ms) for true and false trials for aphantasics and controls in the associated and unassociated conditions

For response-time analyses we included only correct responses. As predicted, there was a significant effect of relatedness on response time to true trials, χ^2^(1) = 3.90, *p* = .048, *R*^2^ = .041, with response time faster in the unassociated (819 ms) compared with the associated condition (956 ms, see Fig. 2). There was no difference between aphantasics and controls in response times for true trials, χ^2^(1) < 1, *p* = .415, *R*^2^ = .005. Contrary to our prediction, there was no interaction between group and relatedness, χ^2^(1) < 1, *p* = .614, *R*^2^ = .001.

We also found a significant effect of relatedness on response time for false trials, χ^2^(1) = 6.67, *p* = .010, *R*^2^ = .056 (where the correct response was “no”), again with responses faster in the unassociated (854 ms) compared with the associated condition (1,054 ms; see Fig. 2). There was no effect of group on response time to false trials, χ^2^(1) = 1.01, *p* = .315, *R*^2^ = .007, and no interaction between group and relatedness, χ^2^(1) < 1, *p* = .414, *R*^2^ = .004. Results for both true and false trials suggest that the effect of relatedness does not differ between aphantasics and controls.

### Accuracy

**Fig. 3:**
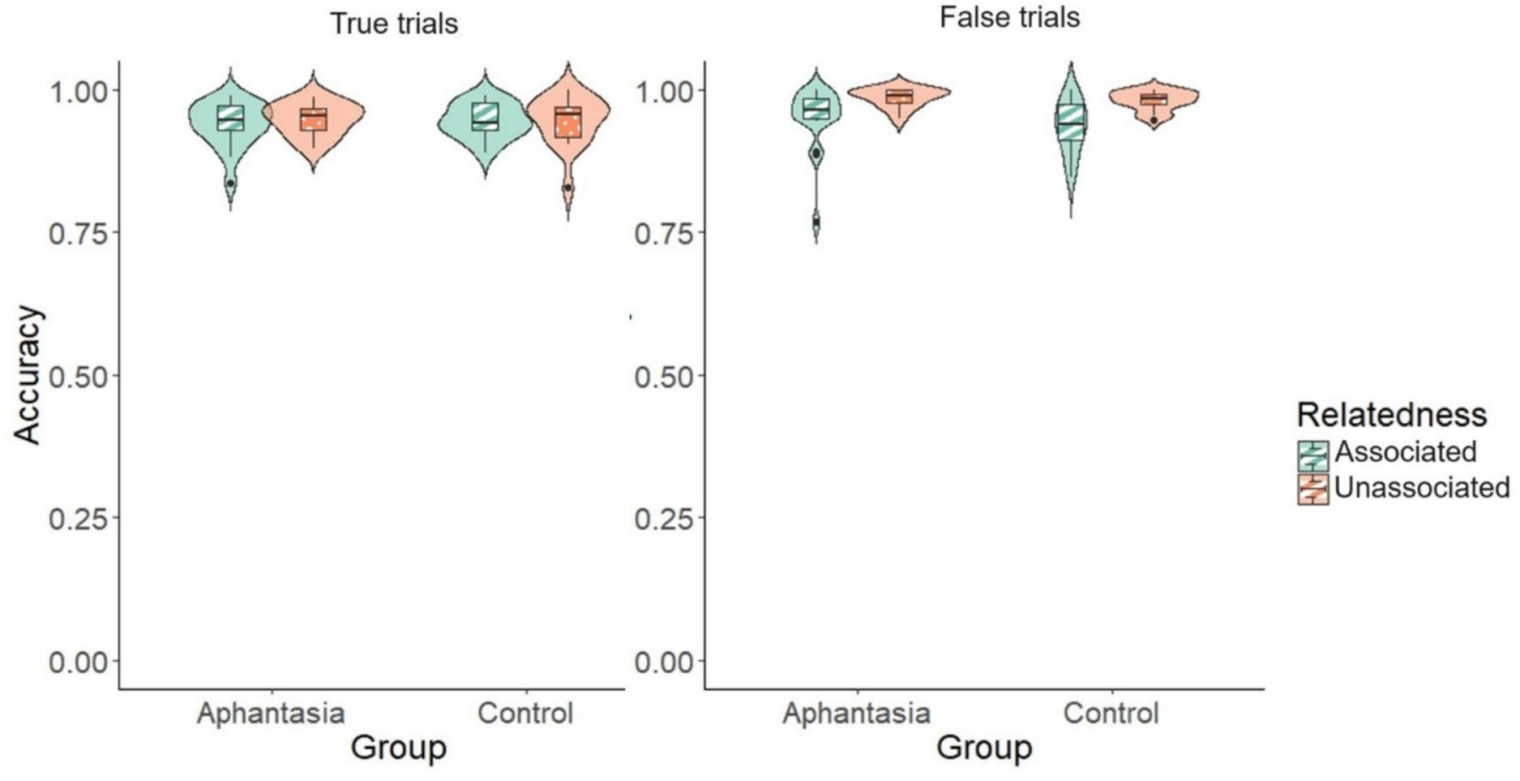
Accuracy (proportion correct) for true and false trials for aphantasics and controls in the associated and unassociated conditions

Models of accuracy did not converge with participants and items modelled as random effects, nor with length and frequency added as covariates, so we report analyses with only participant level intercepts. For true trials there was no effect of group, χ^2^(1) < 1, *p* = .889, *R*^2^ <.001, no effect of relatedness, χ^2^(1) < 1, *p* = .592, *R*^2^ = .00, and no interaction, χ^2^(1) < 1, *p* = .697, *R*^2^ ≤ .001. As shown in Fig. 3, overall accuracy was very high in all conditions. For false trials there was a significant effect of relatedness, χ^2^(1) = 14.80, *p* < .001, *R*^2^ = .005, with accuracy being higher in the unassociated (.98) than the associated condition (.94). There was no effect of group, χ^2^(1) = 2.14, *p* = .4, *R*^2^ = .001, and no interaction, χ^2^(1) < 1, *p* = .9, *R*^2^ < .001.

### Linguistic and perceptual predictors

To analyze the role of the linguistic and perceptual predictors in property verification judgments, we conducted linear mixed effects models of true trials separately for each condition (aphantasic associated, aphantasic unassociated, control associated, control unassociated) with participants and items as random intercepts, and property word length and frequency (Zip frequency) as covariates. This was possible only for response time because the accuracy models did not converge. The unique contribution of perceptual predictors was tested by comparing a full model with all perceptual predictors, the linguistic predictor, and the expectancy predictor, with a model with only the linguistic predictor and expectancy predictor. The unique contribution of the linguistic predictor was tested by comparing a full model of all perceptual predictors, the linguistic predictor, and the expectancy predictor, with a model with only the perceptual predictors and expectancy predictor.

For aphantasics, there was a significant effect of the perceptual predictors over and above the linguistic and expectancy predictor in the associated condition, χ^2^(1) = 17.44, *p* < .001, *R*^2^ = .009, but not the unassociated condition, χ^2^(1) = 9.69, *p* = .085, *R*^2^ = .008, as in Solomon and Barsalou (2004). There was an effect of the linguistic predictor over and above the perceptual and expectancy predictors for the aphantasics in both the associated and unassociated condition, associated condition: χ^2^(1) = 7.68, *p* = .006, *R*^2^ = .004, unassociated condition: χ^2^(1) = 15.29, *p* < .001, *R*^2^ = .013.

For the control group, there was a significant effect of the perceptual predictors over and above the linguistic and expectancy predictor in the associated condition, χ^2^(1) = 13.95, *p* = .016, *R*^2^ = .007, but not the unassociated condition, χ^2^(1) = 7.27, *p* = .201, *R*^2^ = .002. There was an effect of the linguistic predictor over and above the expectancy and perceptual predictors in the unassociated, χ^2^(1) = 11.32, *p* < .001, *R*^2^ = .004, but not the associated condition, χ^2^(1) = 3.77, *p* = .052, *R*^2^ = .002. Results for the control group are in line with the results of Solomon and Barsalou (2004). Table 1 depicts the unique variance accounted for by the perceptual and linguistic predictors in each condition.

**Table 1:** Unique variance in response times explained by perceptual and linguistic predictors using linear mixed-effects analyses (asterisks indicate significant variance explained)

	Aphantasics		Controls	
Predictor	Associated	Unassociated	Associated	Unassociated
Perceptual	.009*	.008	.007*	.002
Linguistic	.004*	.013*	.002	.004*

The analyses suggest that aphantasic individuals do use perceptual simulation in the associated condition, just like controls. Surprisingly, the variance explained by the perceptual predictors is larger for the aphantasics than controls. The results also show that aphantasic participants use linguistic associations in the unassociated condition, again like the control participants. We also see that the aphantasic participants rely on linguistic associations in the associated condition too, while this was not the case for the controls (although *p* = .052).

Solomon and Barsalou (2004) analyzed their data using multiple regression by items. We therefore conducted the same analyses to compare *R*^2^ values with the original study, with property word length and frequency (Zip frequency) as control variables. The pattern of results is the same except that with this analysis, the linguistic predictors explain significant variance in all conditions (see Supplementary Analyses).

As exploratory analyses we examined the unique variance for each individual predictor by comparing full models including all predictors with models without the individual predictor of interest. As shown in Table 2, the effect of the perceptual predictors is primarily driven by P_initial (whether the property was in the initial visual image of the concept) in all associated conditions, as well as the unassociated condition for the aphantasics. Interestingly, in the control unassociated condition, P_area (the area of the property as a percentage of the whole concept) was also a significant predictor, as well as the linguistic variable, L_conc_prop. For both groups then, even when linguistic association is primarily used to make property judgments, perceptual information is to some extent still recruited.

**Table 2:** Unique variance of individual predictors in linear mixed-effects models of reaction time (asterisks indicate significant variance explained)

	Aphantasics				Controls			
	Associated		Unassociated		Associated		Unassociated	
Predictor	β	*R*^2^	β	*R*^2^	β	*R*^2^	β	*R*^2^
*Perceptual*								
P_handle	.052	.002	.008	.000	.047	.002	−.014	.00
P_left	.022	.001	−.031	.001	−.015	.001	.006	.00
P_initial	**−.092**	.004*	**−.105**	.006*	**−.067**	.003*	−.033	.00
P_find	−.019	.00	−.003	.000	.010	.00	−.030	.00
P_area	.052	.002	.013	.000	.036	.001	**.051**	.001*
*Linguistic*								
L_conc_prop	**−.094**	.004*	**−.173**	.013*	−.061	.002	**−.101**	.004*

We also examined the unique variance for each predictor using multiple regression by items, as in Solomon and Barsalou (2004; see Supplementary Analyses). With this analysis the pattern of results differs from the linear mixed-effect models for the control participants in that P_area is no longer a significant predictor in the unassociated condition but only L_conc_prop (the linguistic variable) is, and P_initial is no longer a significant predictor in the associated condition, but P_handle (the likelihood of touching the property) is. This contrast in results demonstrates the importance of considering both participant and item variance in the analysis.

Overall, while perceptual and linguistic predictors to some extent played a role in all conditions, perceptual predictors explained more variance in the associated conditions and linguistic predictors explained more variance in the unassociated conditions, as in the original study. This pattern held for both controls and aphantasics.

## General discussion

We tested the extent to which mental simulation and voluntary mental imagery are related by using an experimental paradigm known to demonstrate mental simulation (Solomon & Barsalou, 2004). We used this paradigm to compare people with low vividness of visual imagery (people with aphantasia) to control participants with intact visual imagery. If mental simulation and voluntary mental imagery are related strongly, one would expect to observe evidence of mental simulation in the control group but not in the aphantasic group.

We found that both aphantasics and controls were affected by the relatedness of the false trials. Responses were faster when the false trials were unassociated compared with associated, presumably because the influence of word association is more rapid than is the influence of mental simulation. Crucially, there was no difference between the two groups of participants. Although we expected aphantasics to be slower than control participants for associated trials in which word association did not reliably cue a “yes” response, the results suggest that they were not hindered by having to rely on mental simulation.

Further evidence from our data supports the suggestion that aphantasic participants used mental simulation to complete the task. As in the original study (Solomon & Barsalou, 2004), perceptual predictors predicted response times when the false trails were associated, and this was true for both groups of participants. This suggests that aphantasics did engage in mental simulation when word association did not reliably cue the response. More broadly this supports the suggestion that mental simulation is flexible and context-dependent, and may not be necessary in all language tasks (Ibañez et al., 2023; Lebois et al., 2015; van Dam et al., 2012).

Our task was similar to that employed by Liu and Bartolomeo (2023), where participants had to judge the similarity of pairs of objects (e.g., beaver, fox) on features such as color and size. In contrast to our findings, however, Liu and Bartolomeo found that aphantasics performed the task more slowly than typical imagers. A crucial difference in their methodology was that participants were explicitly asked to imagine the objects for two seconds each, whereas we did not instruct participants to imagine. The focus on explicit imagery generation may have slowed down information processing. This is in line with Cabbai et al. (2024) who found that imagery could be decoded in V1 in aphantasics when passively listening to evocative sounds, but not when they were explicitly asked to generate imagery of the sound content.

As expected, we found that the linguistic predictor (i.e., the associative strength between the concept and property) predicted response times in the unassociated condition in both groups. Interestingly, the linguistic predictor also predicted response times in the associated condition for the aphantasic participants. While this finding was not expected, it might suggest that aphantasics rely more on linguistic distributional information than do control participants. This could result from a greater reliance on explicit verbal information in everyday thought and memory, to compensate for problems in explicit mental imagery. In line with this idea, aphantasics have been shown to appreciate the language used by a writer in a short story, but appreciate the descriptions of scenery in the short story to a much lesser degree (Speed et al., 2024). An alternative explanation is that aphantasics used linguistic associations because their mental simulation was weak. However, the size of the effect of the perceptual predictors, which is larger than that of the controls, suggests this is not the case.

Our results have important implications for theories of embodied language comprehension because they suggest that mental simulation can be performed independent of strategic or conscious imagery, as previously argued (e.g., Barsalou, 1999). This is consistent with recent evidence showing that aphantasics are less likely to become absorbed in a story world compared with controls with intact visual imagery (Speed et al., 2024). Our results suggest that this kind of story absorption relies more on conscious voluntary imagery than unconscious involuntary simulation. In terms of understanding language at a somewhat basic or superficial level (as in the good enough theory of language comprehension, cf. Ferreira et al., 2002) rather than immersed, enriched story experiences, aphantasics may be able to reactivate visual information as part of word meaning. In fact, Barsalou (1999) already described how unconscious perceptual processing (mental simulation) could underlie cognition without conscious awareness. He stated, “If human knowledge is inherently perceptual, there is no a priori reason it must be represented consciously” (p.583). Since this is one of the first studies to find evidence for mental simulation in aphantasia, the results should be replicated with other mental simulation tasks. One possibility could be to test whether reading words or sentences could lead to visual priming in perceptual tasks such as those observed with binocular rivalry (e.g., Keogh & Pearson, 2018).

Our results also have important implications for theories of aphantasia because they suggest that a specific form of unconscious mental simulation in language processing is preserved in individuals with aphantasia, aligning with previous conjectures (Muraki et al., 2023b). As Krempel and Monzel (2024) argue, however, the involuntary imagery elicited during language comprehension may be only one form of involuntary imagery, so we cannot conclude whether involuntary imagery in general is intact in aphantasia.

It also remains unclear whether voluntary mental imagery and mental simulation differ due to whether they are intentional and voluntary, or whether they occur via conscious or unconscious processes. Because aphantasics are known to consciously experience mental imagery when they dream (Zeman et al., 2020), which is thought to be involuntary, it might be likely that the problem lies in the voluntary generation of imagery (see Nanay, 2021, for a discussion of voluntary vs. involuntary and conscious vs. unconscious visual imagery). In line with the existence of mental simulation in individuals with aphantasia, Cabbai et al. (2024) found decodable sensory representations in V1 in a group of aphantasics during spontaneous imagery (listening to an evocative sound such as a cat meowing), even when the subjective experience of imagery was absent. Interestingly, decoding in V1 was at chance during an explicit imagery task in aphantasics.

An alternative explanation is that aphantasics were not able to visually simulate the meaning of the words to perform the property verification judgement, but used another strategy. This would be in line with the suggestion that cognition is also supported by propositional representations, not only sensory (Pearson & Kosslyn, 2015). This has been suggested as an explanation for aphantasics’ performance on other tasks where an expected impairment was not observed (Scholz, 2024). For example, a single aphantasic participant did not perform more poorly than a group of controls on a mental imagery task, and performed more poorly on a visual working memory task only on very difficult trials (Jacobs et al., 2018). Similarly, a group of aphantasics were not impaired in accuracy on a range of neuropsychological tasks putatively associated with visual imagery (Pounder et al., 2022). Aphantasics have even been found to perform better than controls on visual working memory (Keogh et al., 2021) and mental rotation tasks (Kay et al., 2024). Interestingly, aphantasics reported more often using an analytic strategy (e.g., using logic and rules) to complete a mental rotation task, instead of imagining visual rotation of the objects or rotation of their own body (Kay et al., 2024). In our study it is possible that property verifications were accomplished based on abstract information, rather than mental simulation. For example, verbally generating parts might enable a participant to correctly judge that “sleeve” is a physical part of “blouse.” If this was the case, however, we would not expect perceptual ratings to predict performance. Alternatively, the perceptual ratings may be correlated with another kind of linguistic information not measured by our word association variable, although it is not clear what that would be. On the other hand, the time course of linguistic processing and mental simulation is purported to differ (Barsalou et al., 2008), and would therefore lead to response time differences in the task, which was not observed here. Experimental paradigms that have been used to test the effect of language on visual processing, such as sentence–picture verification tasks (Connell, 2007) or visual discrimination tasks (Meteyard et al., 2007), may provide stronger evidence for mental simulation in aphantasia.

Another possibility is that visual simulation is impaired in aphantasia, but mental simulation in other modalities is spared. When looking at the experimental items, only approximately 60% of the true trials could be answered correctly based on haptic simulation. For example, it is highly likely that participants have no (or perhaps extremely little) haptic experience with the parts of many of the (often wild) animals that serve as stimuli in our study (e.g., cow–udder, pig–snout, shark–fin, zebra–hoof). Furthermore, this also is the case for a number of the nonliving object–part combinations that served as related trials (e.g., church–steeple, sailboat–mast). Therefore, we do not believe that haptic simulation was used as a compensatory strategy by aphantasics for two reasons. If haptic simulation had been used by one group and visual simulation by the other group, a response time difference would still be expected. It has been shown that words’ visual associations typically facilitate word processing, but haptic associations instead slow down word processing (Speed & Brysbaert, 2022). In addition, only five aphantasic participants reported impaired imagery in only the visual modality, whereas 13 reported impairments in all modalities,^2^ suggesting haptic simulation was unlikely.

Another potential limitation to our findings is that we do not have objective evidence that the aphantasic participants lack visual imagery. To determine whether participants have aphantasia, we used the VVIQ (Marks, 1973). This questionnaire requires participants to make subjective judgments on the vividness of their visual imagery when reading short scenarios. Although there do exist some limitations to using a subjective questionnaire, previous research suggests there is a strong relationship between VVIQ scores and more objective measures of aphantasia. Keogh and Pearson (2024) found that 88% of participants with a VVIQ score <32 (the criterion we used here) showed no priming effect in a binocular rivalry task. We are therefore confident that the VVIQ is a good proxy for vividness of visual imagery.

In conclusion, while people with aphantasia are impaired in conscious, voluntary imagery, our results suggest that they can engage in unconscious, involuntary mental simulation during language comprehension. Visual imagery and mental simulation are separable cognitive processes.

## Supplementary Information

Below is the link to the electronic supplementary material.

Supplementary file1 (DOCX 21 KB)

## Data Availability

Data, analysis scripts, and stimuli are available here: https://osf.io/j9znu/

## Footnotes

1 See Appendix for original Dutch translations of rating scales.

2 Seven participants reported impaired imagery in one or more modalities, and five were unsure.
