# Supplementary material for: Dissociating voluntary mental imagery and mental simulation: Evidence from aphantasia
Source: Mem Cognit. 2025 Jun 10;53(8):2674–85. doi: 10.3758/s13421-025-01731-y (PMC12696087; doi:10.3758/s13421-025-01731-y)
Supplement: Supplementary file 1 — Supplementary file1 (DOCX 21 KB) [file 13421_2025_1731_MOESM1_ESM.docx]

**Appendix**

Rating instructions for predictors

L_conc_prop: *Op een schaal van 1 tot 9, hoe snel moest u hieraan denken? (1 = totaal niet, 9 = erg snel)*

P_area: *Stelt u zich een X*^[[1]](#footnote-1)^ *voor. Hoeveel procent van een X is een Y?*

P_left: *Stelt u zich een X voor die zich bevindt in een doos die precies zo groot is dat de/het X alle vier de randen raakt. Hoeveel procent van de hele breedte van de doos is dan de afstand van de linkerkant van de doos naar de/het Y van de/het X?*

P_initial: *Stelt u zich een X voor. Ziet u een Y in uw voorstelling?*

P_find: *Stelt u zich een X voor. Geef op een schaal van 1 tot 9 aan hoe makkelijk u de/het Y van de/het X kunt vinden in uw voorstelling (1 = heel moeilijk, 9 = heel makkelijk).*

P_handle: *Stelt u zich een Y voor. Op een schaal van 1 tot 9, hoe snel zou u hiervan de/het Y aanraken? (1 = niet, 9 = heel snel)*

E_num_obj: *Hoeveel verschillende soorten objecten kent u die (een) X hebben?*

**Supplementary analyses**

Table S1. Pearson correlations between predictor variables

|  | P_area | P_find | P_handle | | P_initial | | P_left | E_num_obj |
| --- | --- | --- | --- | --- | --- | --- | --- | --- |
| L_conc_prop | .310* | .621* | .177 | .508* | | -.238* | | -.169 |
| P_area |  | .328* | .421* | .274* | | -.418* | | -.024 |
| P_find |  |  | .305* | .638* | | -.104 | | .033 |
| P_handle |  |  |  | .155 | | -.305* | | -.028 |
| P_initial |  |  |  |  | | -.171 | | .140 |
| P_left |  |  |  |  | |  | | .006 |
| E_num_obj |  |  |  |  | |  | | 1 |

Analyses conducted as in Solomon and Barsalou 2004

Table S2: Unique variance in response times explained by perceptual and linguistic predictors using multiple regression. Asterisks indicate significant variance explained.

Aphantasics Controls

Predictor Associated Unassociated Associated Unassociated

Perceptual **.145* .108* .166*** .054

Linguistic **.044* .058* .042* .093***

Table S3. Unique variance of individual predictors in multiple regression

|  | Aphantasics | | | | Controls | | | |
| --- | --- | --- | --- | --- | --- | --- | --- | --- |
|  | Associated | | Unassociated | | Associated | | Unassociated | |
| Predictor | β | R^2^ | β | R^2^ | β | R^2^ | β | R^2^ |
| *Perceptual* | | | | | | | | |
| P_handle | .182 | .024 | .011 | .000 | **.239** | **.042*** | -.039 | .001 |
| P_left | .141 | .015 | -.077 | .005 | -.060 | .003 | .071 | .004 |
| P_initial | **-.308** | **.046*** | **-.370** | **.066*** | -.231 | .026 | -.228 | .025 |
| P_find | -.127 | .007 | -.040 | .001 | .037 | .001 | -.030 | .000 |
| P_area | **.215** | **.031*** | .100 | .007 | .211 | .030 | .181 | .022 |
| *Linguistic* | | | | | | | | |
| L_conc_prop | **-.292** | **.044*** | **-.337** | **.058*** | **-.285** | **.042*** | **-.455** | **.093*** |

1. X = concept word, Y = property word [↑](#footnote-ref-1)
